# Dental Department

**Published:** 1899-11

**Authors:** F. S. Caspar

**Affiliations:** Austin, Texas


### Dental Department.

F. S. CASPAR, D. D. S., AUSTIN, TEXAS.

An Ideal Filling.—F. B. Spooner, D. D. S., of Brooklyn, in an article published in the "Items of Interest," says:

"For the last two years I have been experimenting to find the 'ideal' filling so long talked about in our meetings. I do not mean to say it is found, but will give the results of my efforts.

"We all know the essentials of the 'ideal.' Gold costs too much in time and material. Amalgam shrinks, discolors, and is a conductor. Cement is perishable, but ideal in all other respects. Could we get cement to be as lasting as amalgam, what a stride it would be."

Further on the doctor says:

"The conclusion was reached that if I could mix my cement with metal cobblestones, the wash of saliva and other causes of disintegration could be lessened. Mixing my cement with various quantities of alloy, I finally hit on this: On a pad was placed two equal piles of material, one amalgam, the other cement; also acid, q. s. The alloy was first mixed with the liquid. Why? To be sure that the metal becomes thoroughly coated with the liquid. No fear that the cement would finally mingle with the acid, and also form perfect union with the metal. Next, mix the zinc-powder with the wet alloy, working it up to a tolerably stiff mass. It may be said, by the way, that there is much in spatulating cement. It should be so done that all parts will be thoroughly incorporated."

The doctor claims for this filling: First, it is a non-conductor; second, it is adhesive, holding together frail walls. It will not expand, contract or discolor; will not wash or wear away as soon as cement will."

We are satisfied that the combination above described is all that is claimed for it, but we imagine that it will have one trouble not enumerated above, viz.: The cement will eventually wash out from the metal, leaving a rough, granular surface to catch foreign substances. At least, this has been our experience.

A few days ago Dr. T. J. Thomas, late of Bilboa, Spain, showed to me a combination of metal and cement, and demonstrated a new idea of mixing same, which I have tried (so far, to my entire satisfaction and gratification.) If it does, as I fully expect it will do, it will supersede amalgam effectually. The only objection to it, I have found so far, is the color; it presents a steel gray color, which would be unsightly in the front teeth. It has a smooth, shining surface, if, on finishing, the operator uses a ball burnisher, slightly warm. After hardening, it is with great difficulty a drill can penetrate the mass. Indeed, I was so much impressed with this new filling material, that I have tried to persuade Dr. Thomas to place it upon the market, and I think he will do so.

Chloral Camphor.—Dr. A. W. Harlan advises the use of chloral camphor instead of cocaine or other poisonous drugs, to benumb sensitive gums. He says: "Dip a pair of pointed pliers in a solution of chloral-camphor, passing it gently around the root of a tooth which is free from blood or saliva. In a short time you can proceed, with little or no pain,—and you have not a bad smelling drug in the mouth."

EASILY NAUSEATED PATIENTS.—Many people are very sensitive, and will become nauseated at the slightest touch at the back part of the tongue or the roof of the mouth. This can be overcome readily, I presume, in every case, by simply applying a little spirits of camphor to the tongue. A couple of drops will allay that sensation almost instantly, so that you can take your impressions just as nicely as if the patient were not at all sensitive at any time."—Dr. Watkins, in the *International Dental Journal.*

POISON IN TEETH.—This is the style of heading of an article in the St. Louis *Chronicle* of the 6th of October, 1899, and reads as follows:

"Alloy in Filling Thought to Have Caused Death. Xenia, Ohio, Oct. 5 [S. M. T.]. Dr. J. E. Lowery died at his home in Cedarville last night. His death is thought to have been due to an alloy of copper or brass used in filling his teeth by a dentist. His system became filled with poison from the alloy, finally ending in convulsions and death. The fillings were removed shortly before he died.

"This is the second case of poisoning with brass used with material in the mouth, reported from Ohio. The other was in the case of a patient who had some cheap crowns put on by a 'Cheap John Dentist,' and as soon as the gold covering wore off of the brass, the patient became very sick. The result was a suit for heavy damages."

This, of course, seems startling, but we should not be surprised. A competition for cheapness, and not for excellence, seems to pervade the minds of a limited number of the dental profession, and they open their shops, under many headings, but always selecting the name of some large city or country, such as "Chicago Dental Parlors," "New York Dental Parlors," "Columbian Dental Association," etc., etc. Often the unwary will go into these shops, attracted solely by the claims that the "Proprietor" has a claim for public patronage (cheapness) over those who are doing a legitimate practice, and when they come out, they find they have received just what attracted their attention—cheap work, made with cheap material. These men virtually acknowledge by their circulars and signs, their inability to keep pace with other practitioners where skill and science are requisite qualifications, and they adopt this questionable method.

We cannot close this article more appropriately than by quoting Sir Robert Burns: "All works of art must bear a price in proportion to the skill, taste, time, expense, and risk attending their invention and manufacture. They are attended with much less profit to the artist than those which everybody calls cheap. Beautiful forms and compositions are not made by chance, nor can they ever, in any material, be made at small expense."

This expression of the poet covers the case of the dentist.

CARE OF THE HANDS.—Every dentist understands how necessary it is to have clean, smooth hands, soft skin, etc., at this season of the year, when the skin is so liable to chap, rendering the hands unfit to handle delicate instruments, and making them appear unsightly. The following lotion has the curious property of preserving the skin from the effects of cold, preventing chapping and rendering the hands soft, white and smooth:

| | | |
|---|---|---|
| ℞ | Ol. rosæ, gtts........ ...................... | xv |
| | Glycerine ........................... ........ | ℨj |
| | Spirits myrciæ..............................F. | ℨ iij |
| | Ol. cajeputi, gtts.......................... | xx |

M. S.: To be used on the hands every night before retiring, and before going out in the cold air in the morning.

